# Effectiveness and risks of dapagliflozin in treatment for metabolic dysfunction-associated steatotic liver disease with type 2 diabetes: a randomized controlled trial

**DOI:** 10.3389/fmed.2025.1542741

**Published:** 2025-03-25

**Authors:** Hiroo Fukada, Kazuyoshi Kon, Reiko Yaginuma, Akira Uchiyama, Maki Morinaga, Kei Ishizuka, Kyoko Fukuhara, Hironao Okubo, Satoko Suzuki, Shuko Nojiri, Shunhei Yamashina, Kenichi Ikejima

**Affiliations:** ^1^Department of Gastroenterology, Juntendo University School of Medicine, Tokyo, Japan; ^2^Department of Gastroenterology, Juntendo University Nerima Hospital, Tokyo, Japan; ^3^Department of Internal Medicine, Tokyo Metropolitan Tobu Chiiki Hospital, Tokyo, Japan; ^4^Technology Innovation Center, Juntendo University, Tokyo, Japan

**Keywords:** metabolic dysfunction-associated steatohepatitis, sodium–glucose cotransporter 2 inhibitors, vitamin E, tocopherol, sarcopenia

## Abstract

**Introduction:**

Pharmacotherapy for metabolic dysfunction-associated steatotic liver disease (MASLD) is still under development and has not been fully established. For patients with MASLD and type 2 diabetes, treatment with antidiabetic drugs, including sodium–glucose cotransporter 2 (SGLT2) inhibitors, is recommended, with vitamin E supplementation when treatment efficacy is insufficient. The benefits and risks of SGLT2 inhibitors for MASLD with type 2 diabetes have not been thoroughly investigated.

**Objective:**

This prospective randomized controlled trial aimed to elucidate the effectiveness and risks of the SGLT2 inhibitor dapagliflozin in comparison with vitamin E in patients with MASLD and comorbid type 2 diabetes.

**Methods:**

The trial enrolled 24 patients with MASLD and comorbid type 2 diabetes, who were assigned to receive either dapagliflozin (5 mg/day) or vitamin E (150 mg/day) for 24 weeks. The primary outcomes included serum levels of AST, ALT, γ-GT, and type IV collagen, and the FIB-4 index. The secondary outcomes were BMI, HbA1c and serum ferritin levels, lipid profile, body composition assessed using InBody, and hepatic fat content and fibrosis evaluated with FibroScan. Adverse events were monitored throughout the study period.

**Results:**

Both groups demonstrated significant reductions in serum AST and ALT levels but intergroup differences were not significant. The dapagliflozin group showed additional benefits, with significant decreases in BMI and HbA1c, γ-GT, ferritin, LDL cholesterol, and body fat levels, indicating improved glycemic control and lipid profile. Dapagliflozin administration was associated with a significant decline in the skeletal muscle index, indicating a risk of muscle loss absent in the vitamin E group. This reduction in muscle mass is clinically significant as it suggests a potential risk of worsened overall survival with dapagliflozin treatment.

**Conclusion:**

This study indicates that dapagliflozin provides several metabolic benefits in patients with MASLD and comorbid type 2 diabetes, including reductions in the levels of liver enzymes and body fat, but the observed decrease in muscle mass suggests a potential adverse effect on long-term survival outcomes. Muscle mass should be monitored in patients receiving dapagliflozin therapy to mitigate the risk of sarcopenia progression and ensure a comprehensive approach to MASLD management.

**Clinical trial registration:**

https://jrct.niph.go.jp/re/reports/detail/81182, identifier jRCT1031180386.

## 1 Introduction

Metabolic dysfunction-associated steatotic liver disease (MASLD) has become a major global health concern, in view of the burden of chronic liver disease (1–3). Many patients with MASLD present with obesity, and their pathological conditions are closely associated with metabolic syndrome-related diseases such as diabetes, dyslipidemia, and hypertension. Approximately 10% of individuals with MASLD develop a more progressive subtype, metabolic dysfunction-associated steatohepatitis (MASH), which carries an elevated risk of progression to cirrhosis and, in some cases, hepatocellular carcinoma (4). MASLD not only increases the risk of liver disease-related mortality but is also closely associated with the incidence of cerebrocardiovascular events and extrahepatic malignancies, thereby worsening patient prognosis. Establishing pharmacotherapies that effectively improve the prognosis for MASLD is an urgent issue, and various new therapeutic agents are currently under development across different research institutions and organizations. At present, however, MASLD treatment relies on currently available therapies (5).

Type 2 diabetes and MASLD are closely linked through insulin resistance, a pathological state exacerbated by obesity (6). Various antidiabetic medications have been explored for the treatment of MASLD, among which pioglitazone, glucagon-like peptide-1 analogs, and sodium–glucose cotransporter 2 (SGLT2) inhibitors have shown beneficial effects on MASLD pathogenesis. These drugs are recommended in clinical guidelines for the management of the disease in patients with type 2 diabetes (5, 7). SGLT2 inhibitors are antidiabetic agents that lower the levels of blood glucose by inhibiting renal glucose reabsorption, thereby promoting urinary glucose excretion (8). Interventions with dapagliflozin, a highly selective SGLT2 inhibitor, confer multiple beneficial effects, including lowering glucose levels and weight reduction, in clinical studies (9). Additionally, in a single-arm open-label study involving patients with MASH and type 2 diabetes, 24-week administration of dapagliflozin at 5 mg/day significantly reduced the body mass index (BMI) and the levels of serum aspartate transaminase (AST), alanine aminotransferase (ALT), fasting plasma glucose, hemoglobin A1c (HbA1c), and type IV collagen 7S (10). Despite these encouraging results, the benefits and risks of treatment with SGLT2 inhibitors have not been sufficiently investigated.

Vitamin E has been administered to patients with MASLD as an antioxidant, and several studies have demonstrated its efficacy in ameliorating liver damage associated with MASLD (11–14). In a multicenter randomized controlled trial comparing the therapeutic effects of vitamin E and the antidiabetic drug pioglitazone for MASLD, vitamin E was found to be superior to placebo, whereas pioglitazone showed no additional benefit over placebo (15). In Japan, the clinical guidelines for MASLD/MASH recommend administering vitamin E for patients with MASH who do not present with metabolic syndrome or obesity. The guidelines propose that vitamin E be added for patients with MASH and metabolic syndrome-related diseases when drug therapy for syndrome-related diseases alone is insufficient (5).

To date, there is no evidence that SGLT2 inhibitors should be prioritized over vitamin E for the treatment of MASLD in patients with type 2 diabetes. We conducted a multicenter randomized controlled trial to assess the benefits and potential risks of SGLT2 inhibitors compared to vitamin E in the treatment of MASLD complicated by type 2 diabetes.

## 2 Materials and methods

### 2.1 Patient selection

From March 2019 to September 2021, 24 patients with MASLD (five men and 19 women) who provided written and verbal informed consent were enrolled. This study was approved by the Clinical Research Ethics Committee of Juntendo Hospital (CRB3180012) and is registered with the jRCT number jRCT1031180386.

The inclusion criteria were patients with MASLD aged 20–80 years, with HbA1c levels ≥ 6.5%, and evidence of hepatic steatosis on abdominal ultrasound. The exclusion criteria were: (1) alcohol intake exceeding 30 g/day for men or 20 g/day for women; (2) type 1 diabetes; (3) ongoing treatment for liver disease other than MASLD; (4) serum creatinine level ≥ 1.2 mg/dL for men or ≥ 1.0 mg/dL for women; (5) inability to sense thirst (resulting in inadequate self-regulated fluid intake); (6) contraindications to SGLT2 inhibitors; (7) history of hypersensitivity to SGLT2 inhibitors or vitamin E; and (8) any other condition deemed ineligible by a physician.

After obtaining consent and enrollment, the participants were randomly assigned to either the Dapa group or the Vit E group via envelope allocation. The Dapa group received 5 mg/day of dapagliflozin post-breakfast (with an optional increase to 10 mg, as determined by the attending physician), while the Vit E group received 150 mg/day of tocopherol acetate (50 mg three times daily post-meal). Both groups continued the daily medication for 24 weeks. The participants were evaluated at 4, 8, 12, and 24 weeks post-treatment initiation through interviews, blood tests, and analysis of body composition via InBody 770 (InBody Co., Ltd., Seoul, Korea). Additionally, in settings where available, hepatic fat content and stiffness were measured using vibration-controlled transient elastography with FibroScan 502 Touch (Echosens, Paris, France) at baseline and after 24 weeks of treatment (see Supplementary Table 1). Continuation of treatment beyond 24 weeks was optional, with adverse event monitoring extending to 32–48 weeks post-treatment.

### 2.2 Primary endpoints

Primary endpoints were reductions in serum levels of AST, ALT, alkaline phosphatase (ALP), and γ-glutamyl transferase (GT), as indicators of liver injury, and serum type IV collagen and fibrosis-4 (FIB-4) index as markers of fibrosis.

### 2.3 Secondary endpoints

Secondary endpoints included changes in body weight, BMI, and levels of fasting blood glucose and HbA1c, as indicators of glucose tolerance; serum levels of total cholesterol, LDL cholesterol, HDL cholesterol, free fatty acids, and triglycerides, as lipid profile parameters; controlled attenuation parameter (CAP) values and liver stiffness measured via FibroScan; and changes in muscle mass, body water, and body fat measured via InBody.

### 2.4 Statistical analysis

Data entry and aggregation were conducted using the Research Electronic Data Capture (Vanderbilt University, Nashville, TN, USA) platform. The primary analysis followed an intention-to-treat approach for all randomized participants, while safety analysis included participants who had received at least one dose of the study medication. A significance level of 5% and confidence level of 95% were predefined. Intergroup comparisons were performed using ANCOVA with baseline covariates, providing adjusted means, 95% CIs, and *p*-values. Pre–post analysis used paired *t*-tests. The statistical analyses were performed with the SAS version 9.4 software (SAS Institute, Cary, NC, USA).

## 3 Results

### 3.1 Patient characteristics

**FIGURE 1:**
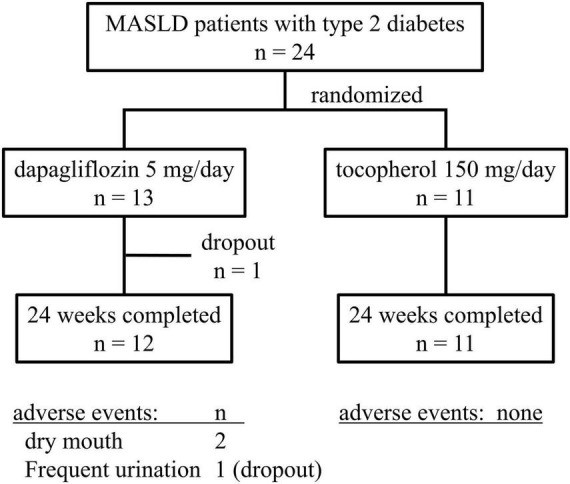
Flow diagram of the process of patient enrollment, randomization, treatment, and follow-up in the study.

The 24 patients enrolled were randomized into two groups, with 13 in the Dapa group and 11 in the Vit E group (Figure 1). Both groups included a higher proportion of female patients. At baseline, four patients in the Dapa group were already receiving statin therapy for dyslipidemia, and in both groups, two patients were being treated with angiotensin II receptor blockers for hypertension. There were no significant differences between the groups for liver enzyme levels, fibrosis markers, or glucose and lipid values. Liver fat changes and stiffness assessed using FibroScan, and body composition using InBody analysis showed no significant intergroup differences (Table 1). None of the patients in the Dapa group required a dose escalation to 10 mg/day.

**TABLE 1:** Baseline characteristics.

	Dapa group		Vit E group		
	**Mean**	**SD**	**Mean**	**SD**	***p*-value**
Age (years)	58.8	16.1	57.6	13.5	0.8435
*Men, *n*	3	23.1	2	18.2	1.0000
BMI (kg/m^2^)	29.5	3.7	30.7	6.5	0.6393
*Statins, *n* (%)	4	30.8	0	0	0.0983
*Pioglitazone, *n* (%)	0	0	0	0	–
*ARB/ACEi, *n* (%)	2	15.4	2	18.2	1.0000
*GLP-1 agonist, *n* (%)	0	0	0	0	–
*SGLT2 inhibitors, *n* (%)	0	0	0	0	–
Platelet count (× 10ł/μL)	20.4	6.2	21.2	7.3	0.7752
AST (U/L)	46.7	16.9	45.9	21.4	0.9228
ALT (U/L)	69.8	53.4	68.5	48.8	0.9504
γ-GT (U/L)	60.7	35.9	55.7	35.3	0.7430
Type IV collagen (ng/mL)	117.2	22.0	142.6	68.1	0.3414
FIB-4 index	2.0	1.1	1.9	1.4	0.8381
Glucose (mg/dL)	138.9	19.8	134.7	22.9	0.6901
HbA1c (%)	6.8	0.4	6.8	0.4	0.9837
Total cholesterol (mg/dL)	205.5	40.3	200.7	27.4	0.7493
LDL cholesterol (mg/dL)	122.6	40.9	130.9	25.6	0.5973
HDL cholesterol (mg/dL)	56.1	14.2	47.7	8.0	0.1286
Triglycerides (mg/dL)	115.7	43.4	143.9	48.2	0.1632
Ferritin (ng/mL)	241.5	134	304.6	283.6	0.5549
CAP value (dB/m)	299.5	27.2	297.4	57.6	0.9216
LSM (kPa)	8.72	5.46	12.1	9.02	0.3396
Muscle mass (kg)	40.7	8.82	43.9	7.1	0.3769
Body water volume (L)	31.6	6.76	34.2	5.4	0.3542
Body fat mass (kg)	29.8	9.0	31.7	12.6	0.7072
SMI (kg/m^2^)	7.2	1.0	7.6	1.2	0.4199
Body fat percentage (%)	40.5	7.6	38.1	9.2	0.5451

Parameters without * were analyzed using an unpaired *t*-test. Parameters with * were analyzed using the chi-square test or Fisher’s exact test. BMI, body mass index; ARB/ACEi, angiotensin II receptor blocker/angiotensin-converting enzyme inhibitor; GLP-1, glucagon-like peptide-1; SGLT2, sodium–glucose cotransporter 2; AST, aspartate transaminase; ALT, alanine aminotransferase; γ-GT, γ-glutamyl transferase; FIB-4 index, fibrosis-4 index; HbA1c, hemoglobin A1c; CAP, controlled attenuation parameter; LSM, liver stiffness measurement; SMI, skeletal muscle index.

### 3.2 Primary and secondary outcomes

**FIGURE 2:**
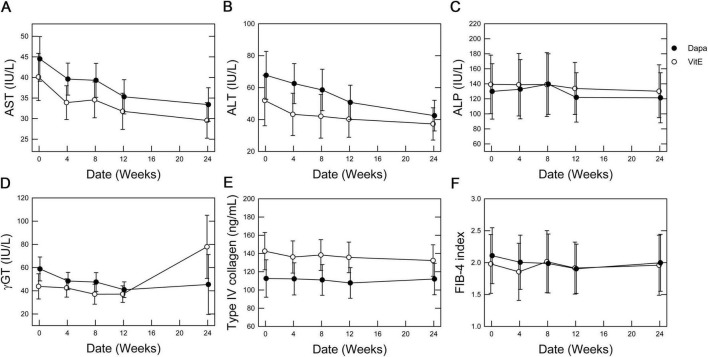
Time-dependent effects of vitamin E vs. dapagliflozin on primary outcome measures. Mean values are plotted with error bars representing the standard error. **(A)** AST levels, **(B)** ALT levels, **(C)** ALP levels, **(D)** γ-GT levels, **(E)** type IV collagen levels, and **(F)** FIB-4 index.

During the 24-week treatment period, a trend of decreasing serum AST and ALT levels was evident in both groups. Serum γ-GT levels decreased only in the Dapa group. No changes were observed in serum ALP or type IV collagen levels, or the FIB-4 index in either group (Figure 2). ANCOVA showed no significant intergroup differences in relation to the primary outcomes (Table 2). For secondary outcomes, the comparison between the Dapa and Vit E groups yielded a *p*-value of 0.0488 for the HbA1c levels, although the 95% CI included zero. No significant intergroup differences were observed in terms of BMI, blood glucose levels, lipid profile, CAP values, liver stiffness measurement (LSM), or body composition (Table 3).

**TABLE 2:** Data analysis and results of primary outcome measures.

	Adjusted mean	95% CI	*p*-value
AST (U/L)	5.39	−3.30, 14.09	0.2105
ALT (U/L)	3.86	−10.38, 18.10	0.5780
ALP (U/L)	0.75	−20.26, 21.75	0.9416
γ-GT (U/L)	−30.25	−101.70, 33.20	0.3012
Type IV collagen (ng/mL)	2.36	−18.33, 23.04	0.8095
FIB-4 index	0.03	−0.25, 0.30	0.8493

AST, aspartate transaminase; ALT, alanine aminotransferase; ALP, alkaline phosphatase; γ-GT, γ-glutamyl transferase; FIB-4 index, fibrosis-4 index.

**TABLE 3:** Data analysis and results of secondary outcome measures.

	Adjusted mean	95% CI	*p*-value
BMI (kg/m^2^)	−0.38	−1.45, 0.70	0.4616
Glucose (mg/dL)	−3.47	−19.42, 12.48	0.6478
HbA1c (%)	−0.28	−0.55, −0.00	0.0488
Total cholesterol (mg/dL)	10.78	−11.58, 33.14	0.3232 (not parallel)
LDL cholesterol (mg/dL)	7.54	−13.03, 28.10	0.4470 (not parallel)
HDL cholesterol (mg/dL)	−1.17	−7.46, 5.12	0.6964
Free fatty acid (μEq/L)	−199.60	−459.94, 60.73	0.1169
Triglycerides (mg/dL)	1.05	−35.08, 37.18	0.9521
CAP score (dB/m)	−24.62	−68.69, 19.45	0.2490
LSM (kPa)	0.17	−3.60, 3.95	0.9220
Muscle mass (kg)	0.00	−0.92, 0.94	0.9848
Body water volume (L)	0.14	−0.75, 1.02	0.7439
Body fat mass (kg)	−0.93	−3.44, 1.58	0.4400

BMI, body mass index; HbA1c, hemoglobin A1c; CAP, controlled attenuation parameter; LSM, liver stiffness measurement.

### 3.3 Pre–post comparison by group

In the pre–post analysis over the 24-week period, both the Dapa and Vit E groups showed significant reductions in serum AST and ALT levels. In the Dapa group, significant reductions were also observed in BMI, levels of serum γ-GT, blood glucose, HbA1c, and ferritin, and in body fat content. Notably, a decreasing trend in muscle mass was observed exclusively in the Dapa group, with a significant reduction in the skeletal muscle index (SMI) (Table 4).

**TABLE 4:** Pre–post comparisons by group.

		Dapa group			Vit E group			
	***N***	**Mean**	**95% CI**	***p*-value**	***N***	**Mean**	**95% CI**	***p*-value**
BMI (kg/m^2^)	9	−0.90	−1.33, −0.47	0.0014	8	−0.74	−2.12, 0.64	0.2462
ALP (U/L)	12	−10.25	−26.55, 6.05	0.1937	11	−11.55	−30.08, 6.99	0.1953
AST (U/L)	12	−10.58	−18.35, −2.82	0.0121	11	−15.82	−28.03, −3.61	0.0162
ALT (U/L)	12	−24.67	−43.68, −5.66	0.0156	11	−27.10	−50.04, −4.14	0.0252
γ-GT (U/L)	11	−16.73	−32.33, −1.12	0.0380	11	20.73	−49.61, 91.07	0.5263
Type IV collagen (ng/mL)	9	−1.89	−12.04, 8.26	0.6791	7	−10.29	−37.14, 16.57	0.3848
FIB-4 index	12	−0.92	−2.26, 0.42	0.1602	11	−1.19	−3.74, 1.36	0.3244
Glucose (mg/dL)	8	−21.75	−32.88, −10.62	0.0024	9	−15.44	−34.94, 4.06	0.1052
HbA1c (%)	11	−0.43	−0.61, −0.24	0.0004	10	−0.20	−0.58, 0.18	0.2678
Total cholesterol (mg/dL)	10	−2.30	−17.53, 12.93	0.7404	10	−11.1	−37.21, 15.01	0.3613
LDL cholesterol (mg/dL)	10	−8.60	−16.48, −0.72	0.0357	8	−19.63	−47.42, 8.17	0.1390
HDL cholesterol (mg/dL)	10	0.80	−2.62, 4.22	0.6094	7	1.14	−4.79, 7.08	0.6540
Free fatty acid (μEq/L)	6	43.5	−337.5, 424.5	0.7809	6	62.17	−291.6, 415.9	0.6704
Triglycerides (mg/dL)	12	11.0	−6.7, 28.7	0.1996	10	2.0	−31.9, 35.9	0.8976
Ferritin (ng/mL)	8	−83.6	−120.0, −47.3	0.0010	8	−73.5	−181.6, 34.63	0.1520
CAP score (dB/m)	7	−15.29	−57.39, 26.82	0.4086	9	6.0	−50.8, 62.8	0.8138
LSM (kPa)	7	−2.47	−6.13, 1.18	0.1491	9	−3.54	−7.10, 0.01	0.0507
Muscle mass (kg)	9	−0.58	−1.17, 0.01	0.0547	8	−0.64	−1.45, 0.18	0.1070
Body water (L)	9	−0.33	−0.88, 0.21	0.1950	8	−0.5	−1.27, 0.27	0.1705
Body fat mass (kg)	9	−1.71	−2.30, −1.12	0.0002	8	−1.18	−4.26, 1.91	0.3980
SMI (kg/m^2^)	9	−0.133	−0.25, −0.01	0.0353	8	2.08	−3.17, 7.32	0.3808
Body fat percentage (%)	9	−2.42	−5.48, 0.64	0.1053	8	−0.99	−6.72, 4.75	0.6961

All analyses were conducted using ANCOVA. BMI, body mass index; AST, aspartate transaminase; ALT, alanine aminotransferase; ALP, alkaline phosphatase; γ-GT, γ-glutamyl transferase; FIB-4 index, fibrosis-4 index; HbA1c: hemoglobin A1c; CAP: controlled attenuation parameter; LSM, liver stiffness measurement; SMI, skeletal muscle index.

### 3.4 Safety evaluation

Adverse events were observed in six patients in the Dapa group and in three in the Vit E group. In the Dapa group, adverse events included polyuria (*n* = 2), thirst (*n* = 2), chest discomfort and redness (*n* = 1), joint pain (*n* = 1), and back pain (*n* = 1). One patient was diagnosed with breast cancer post-enrollment because of a pre-existing lump. In the Vit E group, adverse events included angular cheilitis (*n* = 1), dizziness (*n* = 1), fatigue (*n* = 1), and back pain (*n* = 1), with no severe adverse events reported.

## 4 Discussion

This is the first randomized controlled trial to prospectively compare the effects of an SGLT2 inhibitor and vitamin E in patients with MASLD and type 2 diabetes. Baseline characteristics indicated that both groups had an average BMI of approximately 30 kg/m^2^, consistent with the mild obesity commonly observed in Asian patients with MASLD. The mean serum ALT level was close to 70 U/L and the CAP value exceeded 290 dB/m, indicating that most patients had significant hepatic steatosis. Liver stiffness exceeded 6.0 kPa in both groups, suggesting moderate fibrosis. Additionally, both groups had elevated serum ferritin levels, reflecting a high disease activity in the MASLD population. Regarding body composition, the participants maintained normal body water volume and skeletal muscle mass, though their body fat levels were notably high, suggesting that the groups largely comprised patients with obesity without sarcopenia (Table 1).

Following treatment initiation, both groups demonstrated a gradual reduction in serum AST and ALT levels, indicating a potential improvement in hepatic steatosis and/or liver injury. The decrease in AST levels appeared nearly parallel between the two groups, indicating similar improvements in liver function. Adjusted mean comparisons showed no significant differences between the groups (Table 2). While the serum ALP levels remained largely unchanged, γ-GT levels declined over time in the Dapa group. Since γ-GT is associated with heightened oxidative stress within the liver in MASLD (16), these findings indicate a potential effect of dapagliflozin in reducing hepatic oxidative stress. Meanwhile, indicators of liver fibrosis, such as type IV collagen levels and the FIB-4 index, were only minimally changed in both groups, suggesting that a significant improvement in fibrosis is unlikely over a 24-week period (Figure 2). Further long-term studies are needed to evaluate the effects on liver fibrosis. Among secondary endpoints, the Dapa group demonstrated a reduction in HbA1c levels compared to the Vit E group, with a *p*-value below 0.05. However, as 95% CI crossed zero, this reduction did not reach statistical significance (Table 3).

To clarify the treatment effects within each group, we conducted within-group comparisons of the pre- and post-treatment values (Table 4). Both groups showed a statistically significant decrease in serum AST and ALT levels. Notably, in the Dapa group, there was a trend toward decreased skeletal muscle mass, with a significant reduction in SMI. This result is particularly important, given that this group largely consisted of patients with MASLD who did not present with sarcopenia at baseline and that the intervention lasted only 24 weeks. Previous studies have also suggested that SGLT2 inhibitors may pose a risk of muscle loss due to increased caloric loss (17). The development of sarcopenia, which is characterized by age-related muscle atrophy, is known to be a worsening factor for fibrosis progression in MASLD (18) and a prognostic factor for adverse outcomes in fatty liver disease (19). In our study, the Dapa group also showed a significant reduction in γ-GT and ferritin levels, suggesting additional therapeutic effects. Both γ-GT and ferritin are associated with oxidative stress in MASLD pathology (16, 20), and the significant reduction in these two markers indicates that dapagliflozin may reduce hepatic oxidative stress, underscoring its therapeutic potential in MASLD management to some extent. Furthermore, the Dapa group showed a significant reduction in BMI and HbA1c levels, as expected, and LDL cholesterol levels also decreased owing to improvements in obesity-related factors. However, our findings suggest that treatment with dapagliflozin may induce muscle atrophy, thereby increasing the risk of sarcopenia, which could negatively affect the prognosis of MASLD.

During the study period, three patients in the Dapa group experienced polyuria or thirst, which reflect adverse effects documented in the dapagliflozin prescription information. One patient withdrew from the study due to polyuria (Figure 1). Additionally, one patient with a breast mass noted prior to enrollment was later diagnosed with breast cancer during the study period; the diagnosis was determined to be unrelated to the study intervention.

Establishing effective treatments for MASLD is a global priority, and numerous new drugs are under development. The farnesoid X receptor agonist obeticholic acid demonstrated improvements in fibrosis in a Phase III trial (21), though it was not approved by the FDA due to safety concerns such as pruritus, changes in lipid profiles, gallstones, and rare cases of drug-induced liver injury. Similarly, other farnesoid X receptor agonists failed to receive approval for MASLD (22). The thyroid hormone receptor-β analog *resmetirom* was approved by the FDA in March 2024 after demonstrating reductions in hepatic fat, transaminase levels, and atherogenic dyslipidemia in a Phase III trial involving patients with MASH (23). However, due to its high cost, only a limited subset of patients with MASLD may benefit from its use. The updated 2024 guidelines from the American Association for the Study of Liver Diseases specifically outline *resmetirom* as a new therapy, with recommendations limited to select cases of moderate fibrosis (LSM of 8–15 kPa on vibration-controlled transient elastography or 3.1–4.4 kPa on MR elastography) (24). Consequently, for the majority of patients with MASLD, conventional therapies are expected to remain the primary treatment option in the foreseeable future. Further evaluation is required to determine the effects of a higher dose of dapagliflozin and the potential benefits of combination therapy with dapagliflozin and vitamin E.

This study has some limitations. First, only quantitative assessments of muscle mass were conducted, without qualitative evaluations such as grip strength or gait speed, which are necessary for the diagnosis of sarcopenia. Second, the small sample size may have impacted statistical power, necessitating future studies with a larger number of participants. Additionally, as the cohort was predominantly female, we could not to assess sex differences. Moreover, the high proportion of female participants may have made the decrease in SMI more pronounced, and the effects on male patients should be further investigated in future studies. Lastly, since the evaluation period was relatively short (24 weeks), further studies are needed to assess the long-term effects on the liver fibrosis, sarcopenia, and patient prognosis.

## 5 Conclusion

In this multicenter randomized controlled trial, both dapagliflozin and vitamin E significantly reduced the AST and ALT levels in patients with MASLD and type 2 diabetes, with no significant differences in effect between the two groups. Dapagliflozin can comprehensively treat patients with MASLD and type 2 diabetes and has the advantage of reducing BMI and body fat mass and improving blood glucose levels. However, it is important to note that dapagliflozin may also reduce skeletal muscle mass, potentially increasing the risk of sarcopenia, which could negatively impact the long-term prognosis of MASLD. Future large-scale, long-term studies based on this research are needed to further assess the effects of these two agents on liver fibrosis and sarcopenia.

## Data Availability

The raw data supporting the conclusions of this article will be made available by the authors, without undue reservation.
