# Supplementary material for: Effectiveness and risks of dapagliflozin in treatment for metabolic dysfunction-associated steatotic liver disease with type 2 diabetes: a randomized controlled trial
Source: Front Med (Lausanne). 2025 Mar 25;12:1542741. doi: 10.3389/fmed.2025.1542741 (PMC11975940; doi:10.3389/fmed.2025.1542741)
Supplement: Supplementary file 1 [file Table_1.docx]

|  | **Screening**  **Enrollment** | **Allocation** | **Experimental Intervention** | | | | **Closeout** |
| --- | --- | --- | --- | --- | --- | --- | --- |
| **Visit** | **1** | **2** | **3** | **4** | **5** | **6** | **7** |
| **Week** |  | **0** | **4** | **8** | **12** | **24** | **32-48** |
| Informed consent | X |  |  |  |  |  |  |
| Vital signs | X | X | X | X | X | X |  |
| Routine blood test | X | X | X | X | X | X |  |
| Liver function test | X | X | X | X | X | X |  |
| Urinalysis | X | X | X | X | X | X |  |
| Adverse event assessment |  | X | X | X | X | X | X |

**Supplementary Table 1.** Study Timeline.
